# Evaluation of Ocular and Systemic Oxidative Stress Markers in Patients with Diabetic Retinopathy

**DOI:** 10.3390/life14121588

**Published:** 2024-12-02

**Authors:** Ana Karen López-Contreras, Diana Esperanza Arévalo-Simental, Fermín Paúl Pacheco-Moisés, María Guadalupe Martínez-Ruíz, Cecilia Olvera-Montaño, Ricardo Raúl Robles-Rivera, Sonia Sifuentes-Franco, Tannia Isabel Campos-Bayardo, Selene Guadalupe Huerta-Olvera, Adolfo Daniel Rodríguez-Carrizalez

**Affiliations:** 1 Institute of Clinical and Experimental Therapeutics, Department of Physiology, Health Sciences University Center, University of Guadalajara, Guadalajara 44340, Jalisco, Mexico; ana.lopez3430@alumnos.udg.mx (A.K.L.-C.); maria.mruiz@alumnos.udg.mx (M.G.M.-R.); cecilia.olvera3372@alumnos.udg.mx (C.O.-M.); ricardo.robles3334@alumnos.udg.mx (R.R.R.-R.); tannia.campos@academicos.udg.mx (T.I.C.-B.); 2 Department of Ophthalmology, Hospital Civil de Guadalajara “Fray Antonio Alcalde”, Guadalajara 44280, Jalisco, Mexico; diana.arevalo1673@alumnos.udg.mx; 3 Department of Chemistry, University Centre of Exact and Engineering Sciences, University of Guadalajara, Guadalajara 44430, Jalisco, Mexico; fermin.pacheco@academicos.udg.mx; 4 Department of Health Sciences—Disease as an Individual Process, Tonalá Campus, University of Guadalajara, Tonala 45425, Jalisco, Mexico; sonia.sifuentes@academicos.udg.mx; 5 Medical and Life Sciences Department, La Ciénega University Center, University of Guadalajara, Ocotlan 47810, Jalisco, Mexico; selene.huerta@academicos.udg.mx

**Keywords:** oxidative stress, diabetic retinopathy, aqueous, vitreous

## Abstract

Proliferative diabetic retinopathy (PDR) is the most severe complication of chronic hyperglycaemi stimulates oxidative stress that changes the retinal basement membrane function and provokes neovascularization, macular edema and retinal detachment. But an oxidative–antioxidant biomarker assessment in ocular matrices, such as aqueous humor (AH) and vitreous, might show the oxidative stress (OS) status in the posterior segment. Here, we show a cross-sectional analytical study of 39 patients who had a vitrectomy and assess the levels of different oxidative–antioxidant biomarkers in blood, aqueous and vitreous humor in three groups: diabetes mellitus 2 (DM2) with PDR [DM(+)PDR(+)] (*n* =13), DM2 without PDR [DM(+)PDR(−)] (*n* = 13) and non-DM2 non-PDR [DM(−)PDR(−)] as the control group (*n* = 13). Our finding suggests the presence of oxidative stress in diabetic retinopathy, as evidenced by increased levels of 8-isoprostanes and decreased levels of total antioxidant capacity from stages before the development of diabetic retinopathy. Our results reveal a notable increment in catalase levels in the DM(+)PDR(+) group in blood and vitreous humor. Likewise, we identified that the DM(+)PDR(−) group presents significant levels in 8-IP and SOD in vitreous humor and blood versus aqueous humor. These finding suggest the role of antioxidant enzymes in compensating oxidative stress mechanisms in PDR development.

## 1. Introduction

Diabetic retinopathy (DR) is a serious sight-threatening complication that is closely associated with diabetes mellitus. It is characterized by incessant damage to the blood capillaries of the retina, which is attributed to various alterations caused by high levels of glucose [1]. In diabetic retinopathy due to chronic hyperglycemia, the microvascular damage in the basement membrane of the retina and capillarity thickening increases vascular permeability, tissue ischemia and the release of several angiogenic factors that results in neovascularization, macular edema and retinal detachment [2]. These events are indicators of the progression in proliferative diabetic retinopathy (PDR), which represents one of the most severe complications of diabetes [1,2,3]. Oxidative stress (OS) occurs when the production of reactive oxygen species (ROS) and reactive nitrogen species (RNS) is higher than the antioxidant defenses present in organisms like superoxide dismutase, glutathione peroxidase, catalase and non-enzymatic antioxidants; therefore, the balance between them disappears [4,5,6]. Thus, OS is increased by the activation of secondary signaling pathways mediated by protein kinase C (PKC) and the overactivity of the hexosamine and polyol pathways, being considered one of the factors in the development of DR [6,7].

There are also different microenvironments in the body, where each organ and tissue may have its own environment, including blood and cells. A given biomarker may be present at multiple sites, and its relationship to retinopathy status may vary depending on the site where it is measured [8]. The variety of ocular matrices that can be collected and analyzed to measure biomarkers is wide, but the application of a biomarker at a clinic depends on the type of ocular matrix to be sampled [9]. In humans, aqueous humor (AH) and vitreous humor are the most suitable matrices for assessing biomarkers relevant to posterior segment disorders, such as DR [9,10]. AH is composed mainly of water (99.9%), plus small amounts of sugars, vitamins, proteins along with other nutrients, as well as growth factors and cytokines. It is primarily responsible for maintaining ocular pressure and oxygenating structures such as the cornea and lens [11,12]. The vitreous is located between the lens and the retina, and due to its close contact with the retina, which has high levels of polyunsaturated fatty acids in addition to greater oxygen absorption and glucose oxidation compared to other tissues, it is susceptible to oxidative stress [13], making the passage of radicals possible towards the vitreous body, which, together with its anatomical position, makes it ideal for sampling to reflect biochemical and pathophysiological changes in retinal disease states, including PDR. The vitreous body is mainly composed of water and a meshwork of fine collagen fibrils embedded with dissolved hyaluronan molecules, inorganic salts, lipids, proteins such as albumin, globulins, coagulation proteins and complement factors that have accumulated from blood filtration [14,15]. The aim of this study was to evaluate the ocular and systemic oxidative stress markers in patients with diabetic retinopathy.

## 2. Patients and Methods

This cross-sectional study was approved by the ethics committee of Hospital Civil de Guadalajara “Fray Antonio Alcalde” Guadalajara, Jalisco, Mexico (folio 058/19), and conducted in accordance with the principles of the Declaration of Helsinki. Written informed consent was obtained from all the participants.

### 2.1. Subjects

All participants were recruited from the Department of Ophthalmology, Hospital Civil de Guadalajara Fray Antonio Alcalde in Guadalajara from 1 February 2019 to 31 January 2021.

Inclusion criteria were as follows:

(a) Aged ≥ 18 years

#### 2.1.1. First Group:

(b) Type 2 diabetic patients with proliferative diabetic retinopathy [DM(+)PDR(+)] undergoing vitrectomy for vitreous hemorrhage (VH) or tractional retinal detachment (TRD) or epiretinal membrane and vitreomacular traction (ERM), (c) HbA1c ≤ 9%, (d) systolic blood pressure ≤ 160 mmHg, (e) diastolic blood pressure ≤ 100 mmHg, (f) serum total cholesterol ≤ 250 mg/dL, and (g) blood Triglycerides ≤ 300 mg/dL.

#### 2.1.2. Second Group:

(h) Type 2 diabetic patients without proliferative diabetic retinopathy [DM(+)PDR(−)] undergoing vitrectomy for vitreous hemorrhage (VH) or tractional retinal detachment (TRD) or epiretinal membrane and vitreomacular traction (ERM), (i) HbA1c ≤ 9%, (j) systolic blood pressure ≤ 160 mmHg, (k) diastolic blood pressure ≤ 100 mmHg, (l) serum total cholesterol ≤ 250 mg/dL, and (m) blood triglycerides ≤ 300 mg/dL.

#### 2.1.3. Third Group:

(n) Non-diabetic controls patients without diabetic retinopathy [DM(−)PDR(−)] undergoing vitrectomy for vitreous hemorrhage (VH) or tractional retinal detachment (TRD) or epiretinal membrane and vitreomacular traction (ERM) due to accident and/or ocular trauma, (o) HbA1c ≤ 5.7%, (p) systolic blood pressure < 130 mmHg, (q) diastolic blood pressure < 85 mmHg, (r) serum total cholesterol ≤ 200 mg/dL, and (s) blood triglycerides ≤ 150 mg/dL.

#### 2.1.4. Healthy Volunteer Group:

Healthy age- and sex-matched volunteers were included as a systemic reference for improving the understanding of the biochemical markers.

Non-inclusion criteria were as follows: (a) subjects who had undergone vitreoretinal surgery or laser surgery in the last six months, (b) subjects who received an intravitreal application of antiangiogenics in the last two months, (c) subjects who had taken oral antioxidants above the recommended intake in the last six months, systemic corticosteroid therapy or cytostatic, and all those without controlled cardiovascular status, (d) those who had prior ophthalmic surgeries, intravitreal corticosteroid, or ocular infections, (e) diabetic complications other than diabetic retinopathy, (f) pregnancy or breast-feeding, and (g) acute or chronic kidney disease.

### 2.2. Examination Procedures

The diagnosis of Diabetes mellitus type 2 was based on criteria outlined by the American Diabetes Association in 1997 and clinical history. The evaluation of DR was according to the diagnostic criteria of the American Academy of Ophthalmology 2001 Annual Meeting by retinal examination using an ophthalmoscope by Retina Specialist.

Laboratory tests were performed to determine 8-isoprostanes (8-IP) and nitric oxide (NO) levels as markers of OS, as well as total antioxidant capacity (TAC), catalase, superoxide dismutase and glutathione peroxidase (GPx) activity. All of these were measured in blood, aqueous humor and vitreous humor.

### 2.3. Sample Collection

Blood, AH and vitreous samples were collected from all participants on the day of surgery. Blood samples for serum and plasma analysis (5 mL) were simultaneously collected from the cubital vein, in vials containing EDTA and without it, and were centrifuged at 1200× *g* at room temperature for 15 min. Serum and plasma samples were stored at −80 °C. AH samples (50–100 µL) were collected at the beginning of cataract surgery with a 27-gauge needle on an insulin syringe. Special care was taken to avoid blood contamination. Samples were immediately cooled and stored at −80 °C. Vitreous humor samples (300–400 µL) were taken immediately after setting the trocars and before turning on the infusion system to avoid dilution. Samples were placed in individual Eppendorf tubes, stored at −80 °C. At the time of analysis, all samples were dissolved at room temperature.

### 2.4. 8-Isoprostanes ELISA Assay

The quantification of 8-isoprostanes was performed according to the methodology described by the kit’s manufacturer (8-isoprostanes ELISA kit, Item No. 516351, Cayman chemicals, Ann Arbor, MI, USA). This assay is based on the competition between 8-isoprostanes and an 8-isoprostane-acetylcholinesterase (AChE) conjugate for a limited number of specific rabbit antiserum binding sites. Since the concentration of the 8-isoprostane conjugate was constant while the concentration of 8-isoprostanes in the samples was variable, the amount of the 8-isoprostane conjugate capable of binding to the rabbit antiserum was inversely proportional to the concentration of 8-isoprostanes per sample, and the absorbance measurement was performed at 405–420 nm.

### 2.5. Nitric Oxide (NO) Assay

Before starting the assay, the samples were deproteinized by the addition of zinc sulfate [16]. A colorimetric test was performed according to the instructions of the kit (Nitric Oxide Assay Kit, User Protocol 482650, Calbiochem^®^, San Diego, CA, USA). Eighty five microliters of the standard or sample was placed in wells and 10 μL of nitrate reductase and 10 μL of 2 mM NADH were added. The plate was agitated for 20 min at room temperature, 50 μL of colorant 1 was added and agitated, and 50 μL of colorant 2 was added, agitated at room temperature, and read at 540 nm.

### 2.6. Total Antioxidant Capacity (TAC) Assay

TAC was measured according to the kit’s instructions (Total Antioxidant Power Kit, No. TA02.120530, Oxford Biomedical Research^®^, Rochester Hills, MI, USA) to obtain a concentration in µM Trolox equivalents. Standards and samples were diluted 1:40, and 200 μL aliquots were placed in each well. The plate was read at 450 nm as a reference value, 50 μL of copper solution was added and incubated for 3 min at room temperature, then 50 μL of stop solution was added, and finally, the microplate was read at 450 nm.

### 2.7. Catalase Assay

Catalase was determined according to the kit manufacturer’s instructions (Bioxytech Catalase-520^®^, cat. 21042, OXIS Int, Tampa, FL, USA), where 30 μL of the diluted standard or sample and 500 μL of substrate (10 mM of H_2_O_2_) were added into tubes, samples were incubated for 1 min at room temperature, and 500 μL of the stop reagent was added. The sample/standard was covered and mixed by immersion, and 20 μL of each reagent was added to a microplate. Two milliliters of HRP/reactive chromogen were deposited, mixed in a water bath, and incubated for 10 min at room temperature. The absorbance was read at 520 nm.

### 2.8. Superoxide Dismutase (SOD) Assay

Measure SOD activity according to the kit manufacturer’s instructions (Superoxide dismutase Assay Kit, Item No. 706002, Cayman Chemicals). Start by adding 200 μL of the diluted radical detector and 10 μL of the standard and sample per well. Start the reaction by adding 20 μL of xanthine oxidase to all previously loaded wells. Record the precise time of the start and addition of xanthine oxidase. Mix the microplate for a few seconds and cover with the lid. Incubate in a mixer for 30 min at room temperature and then read the absorbance at 440–460 nm.

### 2.9. Glutathione Peroxidase (GPx) Assay

The measurement of the GPx activity was performed according to the manufacturer’s instructions (Bioxytech GPx-340^®^, cat. 21017, Richmond, CA, USA). The reagent was based on the oxidation of reduced glutathione in the presence of tert-butyl hydroperoxide, glutathione reductase, and NADPH. The decrease in absorbance at 340 nm following substrate addition was recorded; the rate of decrease in absorbance was directly proportional to GPx activity.

### 2.10. Statistical Analysis

Sample size was calculated using a formula to compare means, where *n* of 13 subjects per group was calculated using a 95% confidence interval, to achieve a power of 80% and a level of significance of *p* < 0.05 to detect a difference in means between the test and reference group (117.13 U/Min/Mg severe Non-proliferative diabetic retinopathy group vs. 100.32 U/Min/Mg mild non-proliferative diabetic retinopathy group, respectively) and 14.84 U/Min/Mg of the standard deviation [17].

The distribution of variables was assessed with the Shapiro–Wilk test, where the measured values were mainly non-normally distributed. For a better understanding, the results are expressed as the mean ± standard error of the mean (SEM). The significance of the difference between groups was evaluated using the Mann–Whitney test, and the Kruskal–Wallis test were used to compare values among study groups. Frequencies were determined per category, as well as percentages and the χ2 (or Fisher’s exact) test. All data were computed using an Excel database and SPSS PC for Windows version 26 (Chicago, IL, USA) for the statistical analysis.

## 3. Results

A total of 53 patients were evaluated in the study, and only 39 patients were included, with 13 patients in each study group. As shown in Table 1, there were no significant differences between the ages of the patients in each group, nor in clinical parameters such as heart rate, respiratory rate, weight and height, or in analytes such as glucose, creatinine and cholesterol. However, there were significant differences between patients in the DM2(+)RDP(+) and DM2(+)RDP(−) groups with respect to years with the diagnosis of diabetes, and HbA1c levels were also significantly higher in the DM2(+)RDP(+) and DM2(+)RDP(−) groups compared to the group without diabetes. A fourth group with 13 healthy volunteers was included as a systemic reference for improving the understanding of the biochemical markers: 8-isoprostanes, nitrites/nitrates, total antioxidant capacity, catalase, and superoxide dismutase. See the dotted line in the graphics.

### 3.1. 8-Isoprostanes

Blood levels of 8-IP in the controls were 14.62 ± 1.73 pg/mL. Blood levels increased with the presence of diabetes and retinopathy in all three study groups from 14.88 ± 1.94 to 24.68 ± 3.79 and 21.68 ± 2.77 pg/mL ([DM(−)PDR(−)], [DM(+)PDR(−)] and [DM(+)PDR(+)], respectively). In AH, 8.41 ± 1.27, 4.04 ± 0.37 and 2.63 ± 0.27 pg/mL, and in vitreous humor, 3.59 ± 0.76, 38.90 ± 5.83 and 4.29 ± 0.59 pg/mL were also obtained, respectively, indicating their presence in these matrices. Since 8-IPs are metabolic degradation products of lipoperoxides, these findings suggest cellular and systemic OS.

### 3.2. Nitric Oxide

Levels of the NO catabolites nitrites/nitrates (NOx) in the controls were 11.97 ± 1.22 µM/mL. The NOx levels found in the different biological matrices in the study groups were, in serum, 8.02 ± 1.70, 6.55 ± 0.73 and 5.50 ± 0.60 µM/mL; in AH, 2.72 ± 0.13, 3.01 ± 0.13 and 3.04 ± 0.11 µM/mL; and finally, in vitreous, 3.16 ± 0.29, 2.77 ± 0.12 and 3.62 ± 0.46 µM/mL [DM(−)PDR(−)], [DM(+)PDR(−)] and [DM(+)PDR(+)], respectively (as seen in Table 2). These results suggest nitrosative stress at the ocular and circulatory levels.

### 3.3. Total Antioxidant Capacity

The TAC represents a set of physiological responses to OS composed of enzymes and different molecules. In the controls, the values found in serum were 2.62 ± 0.17 mM, in the study groups at the systemic level, they were 5.22 ± 0.57, 2.86 ± 0.60 and 4.39 ± 0.23 mM, in vitreous humor, they were 3.55 ± 0.47, 3.36 ± 0.19 and 4.31 ± 0.45 mM, and in AH, they were 4.94 ± 0.46, 4.30 ± 0.31 and 4.40 ± 0.35 mM from [DM(−)PDR(−)], [DM(+)PDR(−)] and [DM(+)PDR(+)], respectively.

### 3.4. Catalase

Catalase activity in blood samples was 198.73 ± 2.77, 132.72 ± 5.23 and 285.64 ± 5.40 U/mL, in AH, it was 122.89 ± 2.65, 131.67 ± 1.55 and 148.72 ± 2.82 U/mL, and in the vitreous body, 129. 79 ± 4.57, 109.64 ± 1.23 and 253.96 ± 4.73 U/mL in each study group [DM(−)PDR(−)], [DM(+)PDR(−)] and [DM(+)PDR(+)], respectively, in contrast to the controls (92.39 ± 2.08 U/mL).

### 3.5. Superoxide Dismutase

Superoxide dismutase is a metalloenzyme that catalyzes the dismutation of superoxide anion by decomposing it into hydrogen peroxide and oxygen molecules for catalase and GPx to act. Levels of 5.59 ± 0.51, 4.65 ± 0.18 and 5.43 ± 0.40 U/mL were found in blood samples, in AH, they were 9.02 ± 0.55, 3.59 ± 0.24 and 4.41 ± 0.37 U/mL, and in vitreous humor, they were 9.46 ± 0.53, 16.94 ± 0.39 and 8.54 ± 0.81 U/mL ([DM(−)PDR(−)], [DM(+)PDR(−)] and [DM(+)PDR(+)], respectively) (Table 2). This contrasts with the controls, who exhibited 10.67 ± 1.95 U/mL.

### 3.6. Glutathione Peroxidase

**Figure 1:**
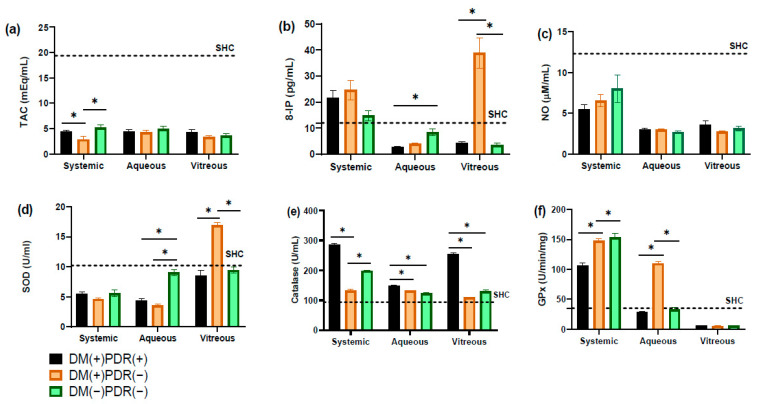
Comparative oxidative stress (OS) biomarkers levels on three study groups, diabetes mellitus 2 (DM2) with PDR [DM(+)PDR(+)], DM2 without PDR [DM(+)PDR(−)] and non-DM2 non-PDR [DM(−)PDR(−)]. OS biomarkers: (**a**) TAC, total antioxidant capacity, (**b**) 8-IP, 8-isoprostane; (**c**) NO, nitric oxide; (**d**) SOD, superoxide dismutase; (**e**) catalase; (**f**) GPx, glutathione peroxidase; SHC, systemic healthy controls. Data are the mean ± SE. * *p* < 0.05.

In the controls, GPx activity was 32.83 ± 3.24 U/min/mg protein. GPx activity decreased in vitreous humor in all groups (5.74 ± 0.49, 5.46 ± 0.14 and 6.37 ± 0.28 U/min/mg), in AH, it oscillated between each group with 32.95 ± 3.72, 110.13 ± 2.46 and 28.88 ± 1.31 U/min/mg, and at the systemic level, there was an increase with 153.03 ± 6.83, 147.36 ± 3.94 and 106.20 ± 4.97 U/min/mg in the [DM(−)PDR(−)], [DM(+)PDR(−)] and [DM(+)PDR(+)] study groups, respectively (Figure 1).

## 4. Discussion

Due to its location, the retina is directly exposed to light and also has a high fatty acid content and high oxygen consumption, which makes it susceptible to OS [18]. There is increasing evidence of the contribution of OS in the pathogenesis of RD [1]. We determined the levels of different markers of oxidant–antioxidant status in blood and aqueous and vitreous humor of patients with PDR and made comparisons between them and with healthy controls. It has been described that the composition of the AH is altered according to the pathophysiology and severity of DR [19]. Also, low-molecular-weight scavengers such as α-tocopherol, glutathione and ascorbic acid are present in the retina, and their mechanisms help minimize OS [20]. 8-IPs are potent vasoconstrictors and act as a mitogen for fibroblasts and vascular smooth muscle cells; these biological actions help explain the reason for the microvascular abnormalities reported in the retinal vasculature in both diabetic and RDP patients, which may contribute to hypoxia and hypoperfusion in the anterior segment of these patients [21,22]. Furthermore, we found increased levels of this biomarker in the three study groups; this also coincides with that reported in other pathologies of ocular origin such as cataracts and exfoliative syndrome [23,24]. Our results reaffirm the findings reported by Lahaie et al. as they propose that 8-IPs contribute to the pathogenesis of retinal ischemia–reperfusion injury, as in the retinopathy of prematurity and diabetes. However, there were no significant differences between the groups. On the contrary, both in aqueous and vitreous humor, we found their levels decreased [25].

The overproduction of NO has been implicated in several pathological processes, from tissue damage together with inflammation and ischemia, followed by cell apoptosis, to something more serious such as acute and chronic neurodegenerative diseases mainly [26]. Kulaksizoglu and Karalezli, in a prospective study with 35 diabetic patients compared with healthy controls who were underwent cataract surgery, found that patients with DM had significantly higher NO levels in the AH, and healthy subjects had significantly higher serum TAC levels. The authors then concluded that the development of PDR is associated with elevated levels of NO in the AH coupled with reduced serum antioxidant defenses [27,28]. The results that we obtained differ from this study because the levels of NO in both aqueous and vitreous humor were lower, and in serum, when compared with healthy controls, there was also a decrement in their concentrations (Table 2), so in the determination of this biomarker, we did not find significant differences between the study groups. More studies are needed to properly understand the effect of NO in the pathogenesis of retinopathy since it is not as simple as saying that the increase or decrease in NO levels is directly related to the severity of the pathology. This is because, according to the findings of different studies, NO concentrations vary according to the area of the retina affected or the severity of diabetes, including the intensity of hypoxia present at the time [26,28].

Each antioxidant system can carry out its activity with a variety of mechanisms and efficacy, according to the chemical structure and stage of the disease. For this reason, TAC is considered an excellent biomarker of the endogenous antioxidant defense system and is widely used since its determination could be much more important than the concentration of individual antioxidants [18]. The levels of TAC in blood, AH and vitreous humor were lower in the group with diabetes but without retinopathy (DM(+)PDR(−)) in comparison to the other two groups; additionally, in blood, there were also lower values compared to healthy controls, which coincides with the behavior of biomarkers reported by Mancino et al. Our results reaffirm that endogenous antioxidant defenses are reduced in patients with diabetes and PDR because their capacity to neutralize oxygen radicals is lower.

Regarding enzymatic antioxidants, SOD activity is very variable in some ocular compartments, even low, as is the case of tears, the aqueous humor and the vitreous body as they are acellular, so the importance of other oxygen radical scavengers must also be considered. Therefore, the eye is almost free of inflammatory reactions, which can be explained by the low levels of SOD isoenzymes [29,30]. This supports the results obtained in our study since the SOD activity found in blood was lower in the three study groups with respect to the healthy controls, Kernell et al. reported that, in non-diabetic patients, total SOD activity was much lower in the anterior chamber, with 9.9 U/mL, than in the vitreous humor, with 106.3 U/mL. We obtained similar results in aqueous humor (9.02 U/mL) but not in the vitreous body (9.46 U/mL). However, the group without diabetes [DM(−)PDR(−)] in the AH had a significant increase in SOD activity compared to the other two groups (*p* < 0.001). In the vitreous body, the group with diabetes but without retinopathy [DM(+)PDR(−)] also had a significant increase in SOD activity relative to the other study groups (*p* < 0.001) [31,32]. Catalase concentrations were higher in the group with retinopathy [DM(+)PDR(+)] compared to the other groups studied in all matrices analyzed; this increase was significant in the aqueous and vitreous humors compared to the other two groups (*p* < 0.001). Information on the determination of catalase at the ocular level is limited; however, currently, antioxidant enzyme activities can be reliably assessed, and the method we used allowed us to effectively detect low concentrations of the enzyme in all matrices [33,34]. On the other hand, in the case of GPx, Huang et al. reported serum concentrations of 3.81(0.84) mg/L (mean(S.D.)) and, in AH, of 0.66(0.18) mg/L in cataract patients. However, there was no significant association between AH and serum levels [35]. Our GPx results obtained in blood were elevated in all study groups with respect to the healthy control, but its detection in AH and vitreous humor was reduced. These values coincide with the results of Pavlovschi et al., who established that a progressive increase in the activity of the GSH system is manifested by an increase in GPx activities [36]. Another important point to take into consideration in the groups without diabetes and without retinopathy that were vitrectomized due to ocular trauma is that this event by itself is proven to generate an increase in oxidative stress, which could explain the behavior of the different biomarkers in this group, where levels were expected to be closer to the healthy groups, but on the contrary, they were comparable to the groups with diabetes [37,38]. The findings so far for all antioxidant enzymes suggest that any imbalance between oxidative and antioxidant defense mechanisms contribute to the development of the proliferative process in diabetic retinopathy [39].

## 5. Conclusions

Our finding suggests the presence of oxidative stress in diabetic retinopathy, as evidenced by increased levels of 8-isoprostanes and decreased levels of total antioxidant capacity from stages before the development of diabetic retinopathy. This indicates that metabolic dysregulation significantly contributes to the depletion of ocular and systemic antioxidant defenses. Consequently, the persistence of this disorder favors the progression of diabetic retinopathy. Determining biomarkers in the vitreous, aqueous humor, and serum enhances our understanding of their behavior, aiming to design a therapeutic strategy that could aid in managing complications associated with diabetic retinopathy. The evaluation of OS biomarkers in matrices such as HA and the vitreous has been standardized, but further studies are still needed to achieve a comparison and to elucidate the role of the antioxidant defense system in PDR.

In terms of the limitations of this study, we accept that it was a cross-sectional study; thus, only one sample was used to decide about the inflammation and OS markers. The levels of the markers do not have static behavior and a follow-up throughout the study with multiple determinations in time is required to corroborate these findings. The study design provides a snapshot of oxidative stress status but does not establish causal relationships or allow for an assessment of disease progression over time.

A cross-sectional study cannot determine the causality between alterations in retina function, OS, and the severity of the disease.

## Figures and Tables

**Table 1:** Demographic data and results of laboratory tests.

	DM2(+)PDR(+)	DM2(+)PDR(−)	DM2(−)PDR(−)	*p*-Value
*n*	13	13	13	*K-W*
Age (years)	55.81 ± 2.64	61.77 ± 2.09	50.92 ± 4.73	NS
Duration of DM (years)	16.18 ± 1.37 †	9.31 ± 1.92 *	N/A	<0.001
SBP (mmHg)	140.59 ± 2.96 ‡	143.00 ± 5.44 ‡	120.23 ± 3.42 *,†	0.001
DBP (mmHg)	85.41 ± 2.06 †	75.31 ± 1.73 *	78.15 ± 2.99	0.004
Heart frequency (b/min)	78.48 ± 2.27	76.85 ± 1.73	77.31 ± 2.86	NS
Respiratory rate (r/min)	17.00 ± 0.44	18.85 ± 1.02	18.54 ± 0.53	NS
Weight (kg)	73.53 ± 3.08	68.19 ± 2.57	79.45 ± 5.13	NS
Height (cm)	160.67 ± 1.86	165.31 ± 3.24	166.69 ± 2.52	NS
BMI (kg/m^2^)	28.41 ± 1.07	24.92 ± 0.69	28.49 ± 1.68	NS
RIOP (mmHg)	14.89 ± 0.86 †,‡	12.61 ± 1.04 *	11.08 ± 0.58 *	0.004
LIOP (mmHg)	16.15 ± 1.50 †	11.00 ± 1.35 *	13.00 ± 0.94	0.009
RVA (LogMar)	1.41 ± 0.20	1.29 ± 0.30	1.47 ± 0.28	NS
LVA (LogMar)	1.62 ± 0.21	1.41 ± 0.29	1.31 ± 0.32	NS
HbA1c (%)	7.39 ± 0.22 ‡	6.54 ± 0.51 ‡	5.20 ± 0.12 *,†	<0.001
Glucose (mg/dL)	112.93 ± 6.35	111.69 ± 4.24	93.81 ± 3.11	NS
Urea (mg/dL)	44.76 ± 3.30 †,‡	27.16 ± 2.95 *	28.25 ± 2.48 *	0.003
Creatinine (mg/dL)	1.48 ± 0.29	0.96 ± 0.17	0.85 ± 0.06	NS
TC (mg/dL)	190.33 ± 9.13	220.41 ± 14.64	181.54 ± 4.49	NS
TG (mg/dL)	193.23 ± 14.88 ‡	214.07 ± 12.62 ‡	106.69 ± 8.34 *,†	0.001

Unless indicated otherwise, data are the mean ± SEM (standard error of the mean). DM, diabetes mellitus; SBP, systolic blood pressure; DBP, diastolic blood pressure; BMI, body mass index; RIOP, right intraocular pressure; LIOP, left intraocular pressure; RVA, right visual acuity; LVA, left visual acuity; TC, total cholesterol; TG, triglycerides. K–W, Kruskal–Wallis test; NS, not significant. *p* < 0.05 between groups: *, [DM2(+)RDP(+)]; †, [DM2(+)RDP(−)]; ‡, [DM2(−)RDP(−)].

**Table 2:** Oxidants and antioxidants.

	DM2(+)PDR(+)	DM2(+)PDR(−)	DM2(−)PDR(−)	*p*-Value
*n*	13	13	13	*K-W*
8-IP (S) (pg/mL)	21.68 ± 2.77	24.68 ± 3.79	14.88 ± 1.94	NS
8-IP (A) (pg/mL)	2.63 ± 0.27 †,‡	4.04 ± 0.37 *,‡	8.41 ± 1.27 *,†	<0.001
8-IP (V) (pg/mL)	4.29 ± 0.59 †	38.90 ± 5.83 *,‡	3.59 ± 0.76 †	<0.001
NO (S) (µM/mL)	5.50 ± 0.60	6.55 ± 0.73	8.02 ± 1.70	NS
NO (A) (µM/mL)	3.04 ± 0.11	3.01 ± 0.13	2.72 ± 0.13	NS
NO (V) (µM/mL)	3.62 ± 0.46	2.77 ± 0.12	3.16 ± 0.29	NS
TAC (S) (mMEq)	4.39 ± 0.23 †	2.86 ± 0.60 *,‡	5.22 ± 0.57 †	<0.001
TAC (A) (mMEq)	4.40 ± 0.35	4.30 ± 0.31	4.94 ± 0.46	NS
TAC (V) (mMEq)	4.31 ± 0.45	3.36 ± 0.19	3.55 ± 0.47	NS
SOD (S) (U/mL)	5.43 ± 0.40	4.65 ± 0.18	5.59 ± 0.51	NS
SOD (A) (U/mL)	4.41 ± 0.37 ‡	3.59 ± 0.24 ‡	9.02 ± 0.55 *,†	<0.001
SOD (V) (U/mL)	8.54 ± 0.81 †	16.94 ± 0.39 *,‡	9.46 ± 0.53 †	<0.001
Catalase (S) (U/mL)	285.64 ± 5.40 †,‡	132.72 ± 5.23 *,‡	198.73 ± 2.77 *,†	<0.001
Catalase (A) (U/mL)	148.72 ± 2.82 †,‡	131.67 ± 1.55 *	122.89 ± 2.65 *	<0.001
Catalase (V) (U/mL)	253.96 ± 4.73 †,‡	109.64 ± 1.23 *	129.79 ± 4.57 *	<0.001
GPx (S) (U/min/mg)	106.20 ± 4.97 †	147.36 ± 3.94 *,‡	153.03 ± 6.83 †	<0.001
GPx (A) (U/min/mg)	28.88 ± 1.31 †	110.13 ± 2.46 *,‡	32.95 ± 3.72 †	<0.001
GPx (V) (U/min/mg)	6.37 ± 0.28	5.46 ± 0.14	5.74 ± 0.49	NS

Values expressed as arithmetic mean ± SEM. S, systemic; A, aqueous; V, vitreous; 8-IP, 8-isoprostane; ON, nitric oxide; TAC, total antioxidant capacity; SOD, superoxide dismutase; GPx, glutathione peroxidase; K–W, Kruskal–Wallis test; NS, not significant. *p* < 0.05 between groups: *, [DM2(+)RDP(+)]; †, [DM2(+)RDP(−)]; ‡, [DM2(−)RDP(−)].

## Data Availability

The data contained in this article are part of the research project registered in Clinical Trials with the identifier NCT04071977.
